# Identification of transcriptional regulatory network associated with response of host epithelial cells to SARS-CoV-2

**DOI:** 10.1038/s41598-021-03309-5

**Published:** 2021-12-14

**Authors:** Chen Su, Simon Rousseau, Amin Emad

**Affiliations:** 1 Department of Electrical and Computer Engineering, McGill University, 755, McConnell Engineering Building, 3480 University Street, Montreal, QC H3A 0E9 Canada; 2 The Meakins-Christie Laboratories at the Research Institute of McGill University Heath Centre (RI-MUHC), McGill University, E M3.2244, 1001 Décarie, Montreal, QC H4A 3J1 Canada; 3 Department of Medicine, Faculty of Medicine, McGill University, Montreal, QC Canada; 4 Mila, Quebec AI Institute, Montreal, QC Canada

**Keywords:** Computational biology and bioinformatics, Gene regulatory networks

## Abstract

Identification of transcriptional regulatory mechanisms and signaling networks involved in the response of host cells to infection by SARS-CoV-2 is a powerful approach that provides a systems biology view of gene expression programs involved in COVID-19 and may enable the identification of novel therapeutic targets and strategies to mitigate the impact of this disease. In this study, our goal was to identify a transcriptional regulatory network that is associated with gene expression changes between samples infected by SARS-CoV-2 and those that are infected by other respiratory viruses to narrow the results on those enriched or specific to SARS-CoV-2. We combined a series of recently developed computational tools to identify transcriptional regulatory mechanisms involved in the response of epithelial cells to infection by SARS-CoV-2, and particularly regulatory mechanisms that are specific to this virus when compared to other viruses. In addition, using network-guided analyses, we identified kinases associated with this network. The results identified pathways associated with regulation of inflammation (MAPK14) and immunity (BTK, MBX) that may contribute to exacerbate organ damage linked with complications of COVID-19. The regulatory network identified herein reflects a combination of known hits and novel candidate pathways supporting the novel computational pipeline presented herein to quickly narrow down promising avenues of investigation when facing an emerging and novel disease such as COVID-19.

## Introduction

Host responses to various insults are coordinated by distinct sets of regulatory networks matching the response to the insult. Viral infections of human cells lead to the production of interferons (IFNs) as an antiviral mechanism^1^. This response must be kept in balance as viral clearance mechanisms can lead to tissue damage if not kept in check^2^. This balance can be especially hard to maintain in the presence of new emerging infections such as the novel coronavirus severe acute respiratory syndrome coronavirus-2 (SARS-CoV-2) responsible for coronavirus disease 2019 (COVID-19), for which the host is naïve. Under those conditions, excessive activation of the immune system can lead to cytokine release syndrome, pulmonary edema, multiorgan failure, and shock^3^, like the severe acute respiratory distress syndrome (ARDS) observed in COVID-19 patients^1^. The TRIF, RIG-I and MDA-5-mediated activation of Interferon response factors (IRFs) is responsible for the expression of antiviral genes, such as type I, II and III IFNs, critical regulators of antiviral immunity. In turn, Type I, II and III interferons activate JAK-STAT signaling to further promote antiviral host responses^4^, which can contribute to the cytokine release syndrome if the response is excessive. Accordingly, JAK-STAT inhibitors have been proposed as attractive drug targets to mitigate the effect of severe COVID-19^5^.

Unraveling the gene expression programs involved in the response of the host to the infection by SARS-CoV-2, by mapping the corresponding transcriptional regulatory network (TRN), can provide a fundamental understanding of COVID-19 and its complications and can enable the identification of therapeutic targets and novel treatments. TRNs represent the regulatory relationships between transcription factors and their target genes, which play significant roles in regulating the gene expression programs in a cell. In this study, our goal was to identify the TRN that was associated with infection by SARS-CoV-2, as opposed to those that are common among infection by different respiratory viruses.

Several studies have attempted building TRNs associated with COVID-19 and the response of the host to the infection by SARS-CoV-2 virus^6–11^. However, none of these studies have incorporated data corresponding to multiple viruses to identify regulatory mechanisms that are enriched or specifically associated with SARS-CoV-2. Moreover, in many of these studies, the TF-gene regulatory relationships are identified based on data corresponding to cancer or normal samples, and not cells infected by SARS-CoV-2^6–9,11^. On the other hand, recognizing the importance of considering the response of host to other respiratory viruses, several studies have sought to identify shared differentially expressed genes, shared pathways or shared gene networks corresponding to multiple respiratory viruses (including SARS-CoV-2)^12–14^.

However, in this study, our goal was to analyze samples infected by multiple respiratory viruses (including SARS-CoV-2) and build a TRN that, while capturing the regulatory influence of TFs on genes, highlights the relationships that are associated with gene expression changes between samples infected by SARS-CoV-2 and those that are infected by other respiratory viruses. Conceptually, such a TRN is different from those constructed only based on samples infected by SARS-CoV-2, those that compare infected samples against uninfected control, or those that try to identify shared mechanisms among the response to different viruses. This TRN would allow us to understand biological mechanisms and gene expression programs that are prominent in the response of host epithelial cells to SARS-CoV-2 that may lead to the identification of biomarkers that are otherwise unidentifiable.

For this purpose, we identified a dataset containing gene expression profiles of human lung epithelial cell lines that were infected by four different respiratory viruses (SARS-CoV-2, RSV, H1N1 and HPIV3)^15^. SARS-CoV-2 is a positive-sense single-stranded RNA virus, part of the broad *coronaviridae* family. Similar to SARS-Cov-1 and the Middle East respiratory syndrome (MERS), SARS-CoV-2 can cause severe acute respiratory disease in humans^1^. Respiratory syncytial virus (RSV) is a single-stranded negative-sense virus, a common cause of mostly mild respiratory disease in children part of the *pneumomviridae* family. However, both in children^16^ and adults^2^, it can lead to serious lung diseases, including ARDS. The influenza A virus H1N1, a negative-sense RNA virus member of the *orthomyxoviridae* family, was responsible for the 2009 swine flu pandemic. Human parainfluenza viruses (HPIV) are negative-sense RNA viruses that cause lower respiratory infections in children, chronically ill and elderly patients^17^ and members of the *paramyxoviridae* family. This dataset is particularly valuable for our study, since it contains gene expression profiles of multiple human lung cell lines infected by four different respiratory viruses, measured using the same gene expression profiling protocol by the same lab. As a result, this dataset enables achieving the goals of this study without introducing confounders such as different protocols or different labs that may distort the analysis.

The main challenge in reconstructing TRNs associated with gene expression changes between samples infected by SARS-CoV-2 and samples infected by other respiratory viruses is how to systematically integrate the association between gene expression data and the virus type used for infection in the TRN reconstruction analysis. Recently, we developed and extensively validated a new computational algorithm called InPheRNo^18^ to integrate sample-level phenotypic or clinical labels (e.g., virus type in this study) and scores (e.g., ln(IC50) scores in pharmacogenomics studies) in the TRN reconstruction procedure using gene expression datasets and identify ‘phenotype-relevant’ TRNs (in this terminology, phenotype refers to sample-level labels/scores mentioned above).

**Figure 1:**
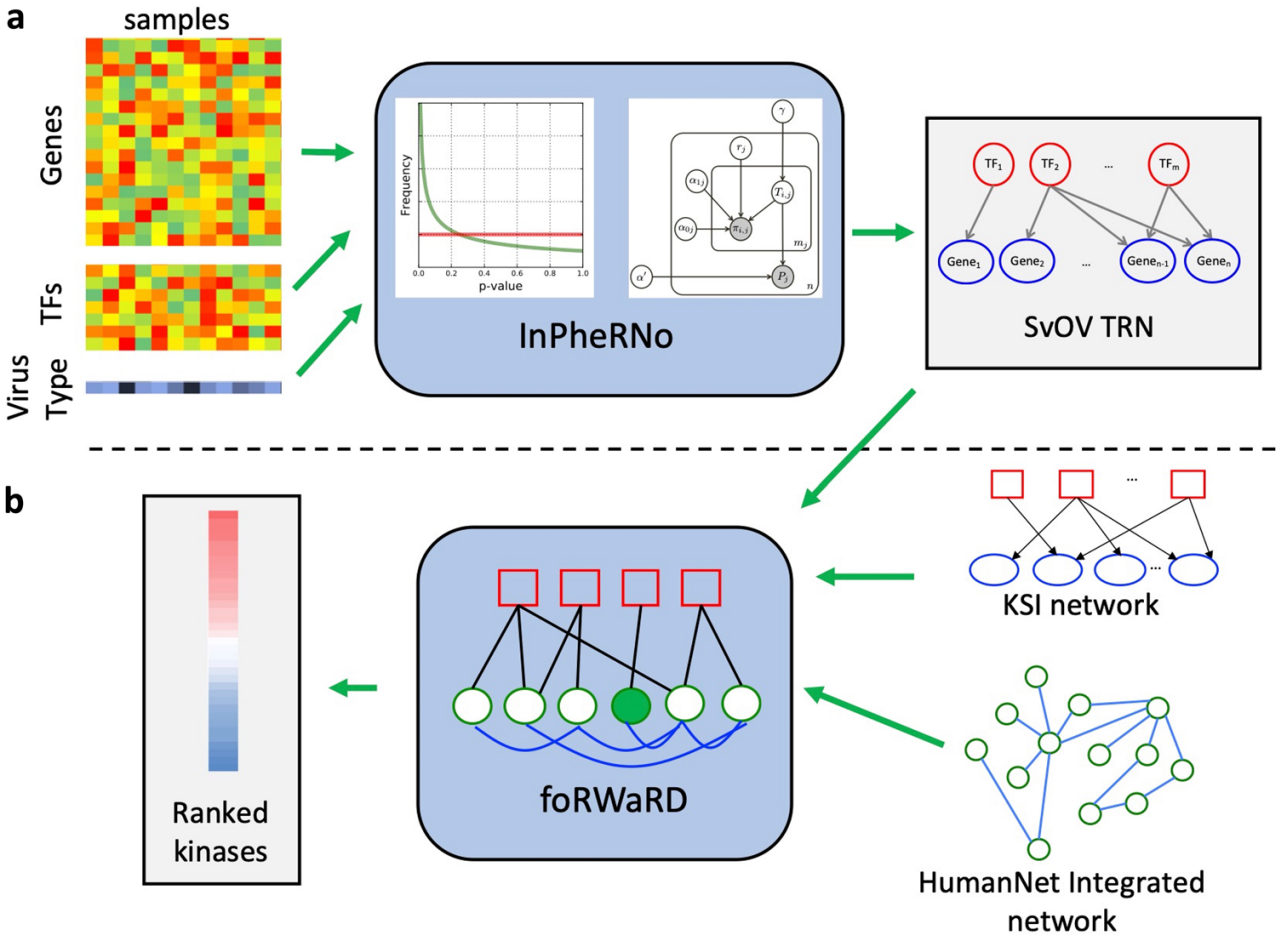
The computational pipeline of this study. (**A**) Differential expression analysis is performed to obtain p-values for the differential expression of the genes in samples infected by SARS-CoV-2 versus samples infected by other viruses. These p-values, along with the RNA-seq expression profiles of all samples, are provided as inputs to InPheRNo. InPheRNo, models the distribution of these p-values as well as the p-values of gene-TF associations obtained using a two-step procedure. Using a probabilistic graphical model, InPheRNo then assigns a score to each possible edge and utilizes them to reconstruct a SvOV TRN. (**B**) The top TFs (those with the greatest number of identified targets in the SvOV network), along with a kinase-substrate interaction network (constructed by us from three studies) and the HumanNet integrated network of gene–gene interactions, are provided as inputs to foRWaRD. foRWaRD then integrates these inputs into a heterogenous network and utilizes a random walk with restarts (RWR) algorithm to assign a ‘ratio score’ to each kinase based on its network proximity to the input top TFs and form a ranked list.

First, using InPheRNo, we reconstructed a TRN associated with gene expression changes between samples infected by SARS-CoV-2 and those that were infected by other respiratory viruses (called SvOV, henceforth) (Fig. 1A). Our results identified known and novel candidate regulatory TFs and signaling pathways involved in response to SARS-CoV-2 and its associated complications. Next, using a network-guided approach based on random walks on graphs, we identified kinases that are most associated with the reconstructed SvOV TRN, as potential regulators of the response of host to infection by SARS-CoV-2 and potential therapeutic targets (Fig. 1B). Kinases are enzymes that are involved in the regulation of protein activities through phosphorylation and are a major category of drug targets for human diseases. Using data from gene knockdown experiments from the LINCS database^19^, we observed that these kinases indeed influence the expression of genes in the reconstructed TRN in epithelial cells. Our analyses using network-based algorithms and machine learning tools provided a systems biology perspective of the response of the epithelial cells to infection by SARA-CoV-2 and identified regulatory mechanisms specific to this virus. In addition, our results implicated important families of kinases (including JAK and MAPK family) that may be used as therapeutic targets for COVID-19.

## Results

### SvOV TRN implicate major transcription factors involved in SARS-CoV-2 host response to infection

We sought to identify transcriptional regulatory mechanisms that are involved in the host response to SARS-CoV-2 infection, but also distinguish this response from response to other respiratory viruses (SARS-CoV-2 versus other viruses, or SvOV). For this purpose, we obtained gene expression profiles of human lung epithelial cells that were infected by SARS-CoV-2, respiratory syncytial virus (RSV), human parainfluenza virus type 3 (HPIV3), influenza A/Puerto Rico/8/1934 (H1N1) virus (IAV), and IAV that lacks the NS1 protein (IAVdNS1) from a recent study^15^. Details of the data are provided in “Methods”.

To reconstruct the SvOV TRN, we used InPheRNo^18^, a method that we recently developed to identify ‘phenotype-relevant’ TRNs using gene expression profiles of multiple samples and their phenotypic labels (in this study, the phenotypic label of a sample corresponds to the specific virus used for its infection). InPheRNo is based on a probabilistic graphical model (PGM) designed to integrate the collective regulatory influence of multiple TFs on a gene with the association of the gene’s expression with a phenotype to identify regulatory mechanisms that are phenotype-relevant (as opposed to phenotype-independent). In this approach, first, the p-values of gene-phenotype associations (e.g., using differential expression analysis) and p-values of gene-TF associations (using a two-step procedure based on the Elastic Net algorithm) are obtained and provided as input ‘observed variables’ to the PGM. The PGM is then trained on the data to obtain posterior probabilities for each TF-gene pair determining whether the TF regulates the gene in a phenotype-relevant manner (details are provided in the original manuscript^18^).

Using data corresponding to SARS-CoV-2 infected samples and samples infected by the other viruses mentioned above, we first performed differential expression analysis (using EdgeR^20^) and then reconstructed the SvOV TRN based on the top 500 differentially expressed genes (false discovery rate (FDR) < 1.43E−3, Supplementary Table S1) using InPheRNo (Fig. 1A). The details of the analysis are provided in “Methods”, and the reconstructed network is provided in Supplementary Table S2. Given the SvOV TRN, we next ranked TFs based on the number of target genes associated with infection by SARS-CoV-2. Table 1 shows the ranked list of the top 21 TFs that target at least 1% of the considered differentially expressed genes in the SvOV TRN (see Supplementary Table S3 for the full ranked list). Encouragingly, some of the TFs identified by InPheRNo have been previously shown to be activated during COVID-19, including the top 2 TFs, STAT1^21^ and STAT2^22^, as well as some other well-established TFs such as TP53^23^ or IRF9^24^.

**Table 1:** Top 21 TFs implicated in the SvOV (SARS-CoV-2 vs. other viruses) TRN.

Transcription factors	Percent of target genes (%)
STAT1	5.89
STAT2	2.95
MLX	2.74
EGR4	1.47
RCOR1	1.47
SP140L	1.47
TP53	1.26
RCOR2	1.26
MAX	1.26
ZNF496	1.26
ZNF512B	1.05
SMAD7	1.05
SOX12	1.05
IRF2	1.05
HDX	1.05
EGR1	1.05
SP110	1.05
IRF9	1.05
ZNF143	1.05
NFIX	1.05
ZBTB32	1.05

The TFs are ranked based on the number of differentially expressed target genes identified by InPheRNo. The second column shows the percent of the considered genes that each TF regulates. As a reference for comparison, the average percent of target genes for a TF is only 0.078% in the SvOV network.

Since our analysis seeks to pinpoint regulatory relationships that are specific to SARS-CoV-2, a systematic evaluation of the results is difficult, since most of the comprehensive databases of TF-gene relationships are formed by integrating data from many different conditions that may not reflect the response of host to SARS-CoV-2 (i.e., they reflect the ‘global’ regulatory mechanisms in a cell). However, one can still expect an enrichment of the identified edges in the SvOV network with such global TRNs. To assess this enrichment, we compared the targets of these TFs identified by InPheRNo, with their targets determined using ChIP-seq data available in the Gene Transcription Regulation Database (GTRD) database^25^. Four of the top TFs in the SvOV network (STAT1, RCOR1, EGR1, ZNF512B) were present in the GTRD dataset, which we used for the evaluation of our network. Out of the 45 targets found for these TFs by InPheRNo, 37 were confirmed using GTRD (p = 2.36E−15, hypergeometric test), showing a high degree of overlap between the SvOV TRN (identified using RNA-seq) and the global TRN (identified using ChIP-seq).

### Functional characterization of the SvOV TRN implicate major signaling pathways involved in COVID-19

In order to determine the functional characteristics of gene expression programs involved in the response of host to SARS-CoV-2, we performed pathway enrichment analysis for the implicated TFs and their identified target genes in the SvOV network. For this purpose, we used the gene set characterization (GSC) computational pipeline of KnowEnG (Knowledge Engine for Genomics) analytical platform^26^, which enables ‘standard’ gene-set enrichment analysis (using Fisher’s exact test), as well as advanced ‘network-guided’ analysis (using a user-selected gene interaction network).

**Figure 2:**
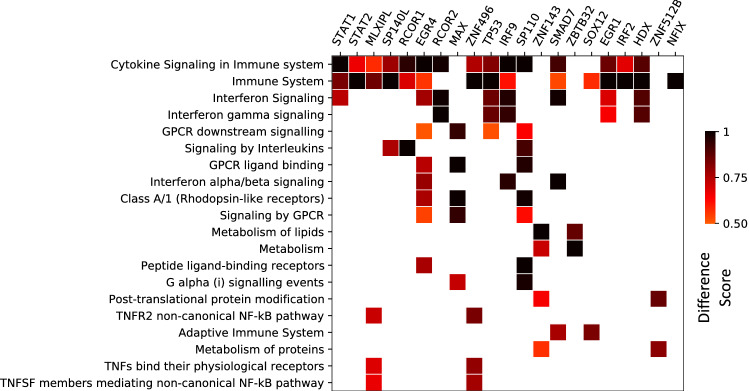
Pathway enrichment analysis using network-guided gene set characterization pipeline of KnowEnG. The columns correspond to top TFs and their targets identified using InPheRNo. Only pathways that have been implicated for at least two TFs (and their targets) are depicted (see Supplementary Table S5 for the full list). The heatmap shows the ‘difference score’ that is used by KnowEnG’s network-guided GSC pipeline to identify associated pathways. A score above 0.5 shows that the pathway is associated with the input query set. In the figure, cases in which the difference score was smaller than 0.5 are shown as white.

To this end, we used the set of top TFs in the SvOV networks (Table 1) as a query gene set and performed standard and network-guided (using experimentally verified PPI network from STRING database^27^) pathway enrichment analysis (using Reactome pathways^28^) with default parameters (Supplementary Table S4). Pathways related to cytokine signaling and interferon signaling (interferon gamma signaling and interferon alpha/beta signaling) were implicated using both standard and network-guided analysis. Next, we repeated the network-guided analysis above by considering each TF and the set of its target genes in the SvOV network as a separate query gene set (Fig. 2 and Supplementary Table S5). Figure 2 shows gene sets that are implicated for at least two TFs and their targets in this TRN. Once again, pathways related to Immune system, cytokines and interferon signaling were again among the pathways implicated for the majority of TFs (and their identified targets).

### Identification of kinases associated with SvOV TRN as potential therapeutic targets

Kinases are enzymes that are involved in the regulation of protein activities through phosphorylation and are a major category of drug targets for human diseases^29^. Consequently, we sought to identify human kinases that are most associated with the constructed SvOV TRN as important signal transducers of infection by the SARS-CoV-2 virus. For this purpose, we formed a kinase-substrate interaction (KSI) network by aggregating kinase-substrate relationships from three previous studies^30–32^ (see “Methods” for details). The aggregated KSI contained 29,594 kinase-substrate relationships corresponding to 406 unique kinases and 3942 unique substrates.

**Figure 3:**
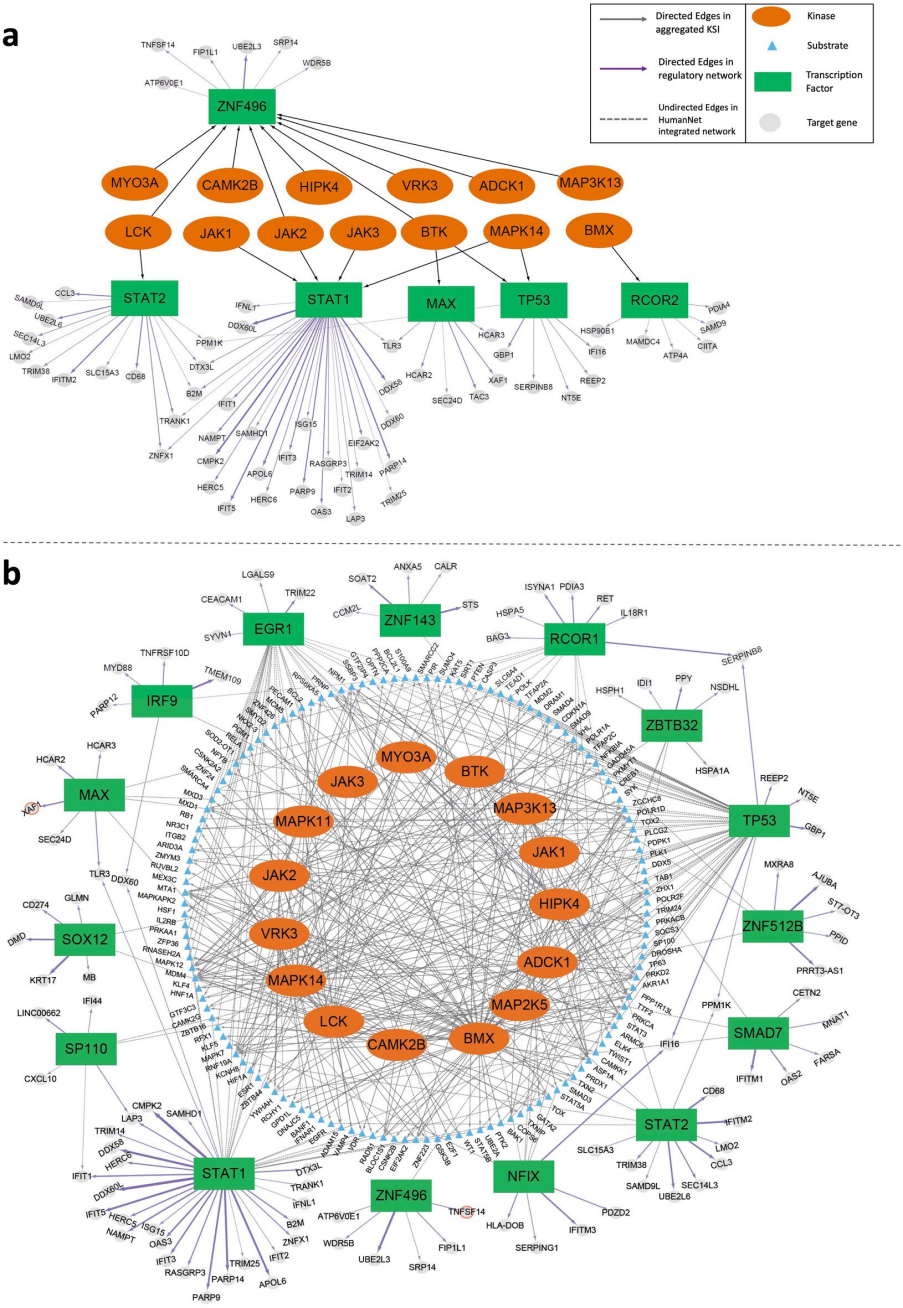
Network representation of implicated kinases, TFs, and their target genes in the SvOV TRN. Kinases are depicted as orange ellipses, TFs are depicted as green rectangles and target genes are depicted as grey ellipses. The width of TF-gene edges is proportional to their score obtained by InPheRNo. The figure was drawn using Cytoscape^62^ version 3.8.2 (https://github.com/cytoscape/cytoscape/releases/3.8.2/). (**A**) This network shows only the direct kinase-TF interactions present in the aggregated KSI. (**B**) This network shows the indirect interactions between implicated kinases, substrates, TFs and their target genes. Substrates that interact with at least one of the implicated TFs in the HumanNet integrated network are depicted as blue ellipses. Directed edges show interactions between kinases and their substrates (obtained from the aggregated KSI) as well as TFs and their target genes (obtained using InPheRNo, purple). Undirected edges correspond to interactions between substrates and TFs (obtained from HumanNet Integrated network).

We used foRWaRD, a computational tool that we recently developed to rank nodes and sets of nodes in a heterogenous network based on their relevance to a set of query set using random walk with restarts (RWR)^33^, to identify kinases associated with the SvOV network. As input to foRWaRD, we provided the aggregated KSI, a gene–gene interaction network (here we used HumanNet integrated network^34^), and a query set containing the top TFs obtained from the SvOV TRN (21 TFs) (Fig. 1B). Table 2 shows the 15 highest ranked kinases for the top TFs in the SvOV TRN and the full ranked lists of kinases are provided in Supplementary Table S6. Figure 3 shows network representations of the interactions among these kinases, their substrates, and the SvOV TRN. Figure 3A only includes direct kinase-TF interactions, while Fig. 3B and Supplementary Table S7 includes indirect interactions of kinases and TFs.

**Table 2:** Top 15 kinases identified using foRWaRD for the top TFs in the SvOV network.

Ranked list of kinases (based on top 21 TFs in SvOV network)	
Kinase	Ratio score
MYO3A	11.60
JAK3	10.74
VRK3	10.34
ADCK1	9.74
JAK1	8.69
MAP2K5	8.19
BMX	7.97
HIPK4	7.48
JAK2	7.46
MAP3K13	7.34
LCK	6.63
CAMK2B	6.34
MAPK14	6.32
BTK	6.13
MAPK11	6.05

The kinases are ranked based on the ratio of their query set probability score to their background probability score. Any ratio score > 1 implies that the kinase is scored higher using the top TF query set compared to its control, with a higher score reflecting a high degree of network proximity to the query set.

In a recent study that evaluated the global phosphorylation landscape of SARS-CoV-2 infection^35^, the activity of a subset of human kinases was evaluated, with kinases related to the p38 family found to be the most up-regulated. Interestingly, the results in Table 2 predicts that p38-related kinases (MAPK11, MAPK14, CAMK2B) form a cluster of kinases regulating SARS-CoV-2 specific TRNs, providing independent experimental evidence that supports the prediction of the model proposed herein.

### Evaluation of the predicted kinase-gene relationships using gene knockdown experiments

Since foRWaRD incorporates both direct and indirect interactions to identify kinases, we sought to determine whether the knockdown of identified kinases directly influences the expression of the TFs and their target genes in the SvOV TRN. To this end, we obtained gene expression signatures corresponding to shRNA knockdown experiments from the LINCS database^19^. We only focused on experiments performed in A549 cell line, since it is one of the cell lines used in our analysis to construct the SvOV TRN. The gene expression signatures correspond to z-score normalized changes in the expression of 978 ‘L1000 landmark genes’ as a result of knockdown of a single gene, when compared to control (no knockdown). For our evaluations, we only used the 978 landmark genes, since these are the only genes that are experimentally measured in the LINCS database, while the other genes are computationally predicted. Since this database does not provide p-values of differential gene expression, we used a threshold-based approach to select genes that are influenced by the kinase knockdown. We defined a landmark gene to be positively (negatively) influenced by the knockdown of a kinase if its expression increased (decreased) as a result of the knockdown and also if its normalized expression change (captured in the signature) was among the top (bottom) 15% of all landmark genes.

**Figure 4:**
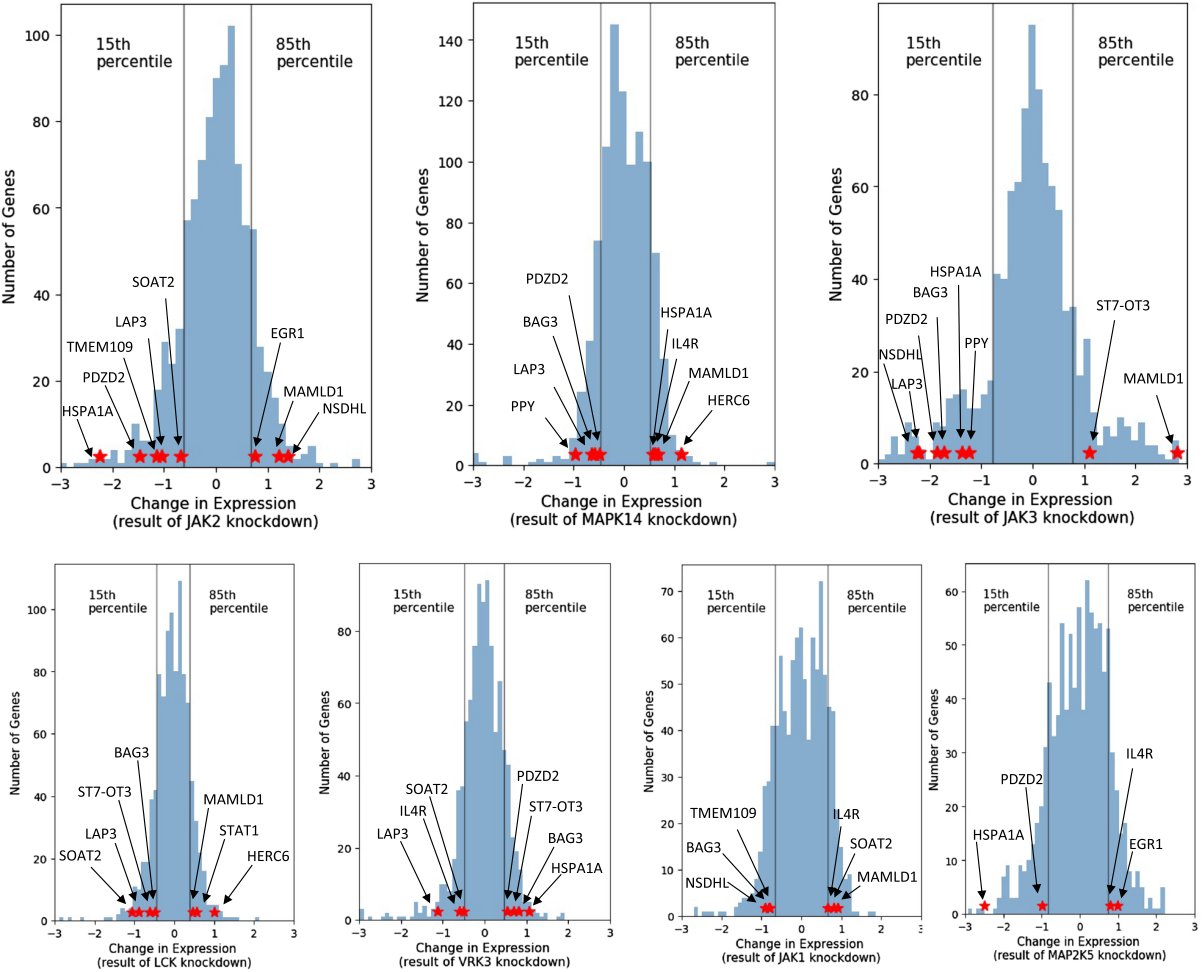
The histogram of z-score normalized gene expression changes of LINCS L1000 landmark genes in A549 cells as a result of knockdown of kinases implicated in the SvOV analysis. Each histogram corresponds to the knockdown of one kinase. Vertical lines depict the 15th and 85th percentiles and red stars show the TFs and target genes in the SvOV network that are positively or negatively influenced by the experiment.

Knockdown signatures were only available for 7 (out of 15) implicated kinases in Table 2. In addition, only 14 target genes and 3 TFs from the SvOV TRN were among the L1000 landmark genes. Our results showed that 12 target genes (out of 14) and 2 TFs (out of 3) were positively or negatively influenced by the knockdown of at least one of the 7 kinases (Fig. 4, Supplementary Fig. S1 and Supplementary Table S8). Figure 4 shows the histogram of the expression changes of the landmark genes as a result of each kinase knockdown and its effect on the TFs and target genes in the SvOV TRN. Taken together, these results suggest that our computational pipeline that strings together InPheRNo, KnowEnG’s GSC, and foRWaRD is capable of identifying biologically plausible signaling networks involved in regulating the response of airway epithelial cells to SARS-CoV-2.

## Discussion and conclusion

Investigation of the regulatory networks specifically associated with SARS-CoV-2 can help better understand either acute complications or long-term consequences of COVID-19. This is particularly important for a novel disease, for which limited information is available. Considering that as of June 2021, more than 176 million people have been infected, the potential of diverging responses is huge and can have a long-lasting impact on the health of many. Therefore, better understanding the peculiarities of SARS-CoV-2 compared to other viruses is paramount to deal with the coming fall out of the pandemic. The aim of this study was to identify such signaling and transcriptional regulatory networks that play key roles in transducing signals specific to SARS-CoV-2 infection of airway epithelial cells in order to better understand the pathophysiology of COVID-19 and provide a list of potential molecular targets for therapies aimed at altering the clinical courses of the disease. The response was studied in comparison to other viruses (RSV, H1N1, HPIV3), which allows to pinpoint mechanisms that are specific to SARS-CoV-2. A systematic comparison with known regulatory mechanisms based on ChIP-seq data confirmed the regulatory relationships between the identified TFs and their target genes. In addition, pathway enrichment analysis identified immune system, cytokines and interferon signaling pathways associated with the reconstructed TRN.

Using a kinase-substrate network, which we formed by aggregating data from three separate studies, we identified several families of kinases associated with the SvOV network (Table 2). JAK1, JAK2, and JAK3, which are identified among the top 15 kinases, belong to the Janus kinase family, a family of non-receptor tyrosine kinases^36^. This family of kinases is involved in the transduction of cytokine-mediated signals through the JAK-STAT pathway. The members of the Janus family and the JAK-STAT pathway have been suggested as potential therapeutic targets in COVID-19^5,37–39^, supporting the findings of our analysis. Additionally, published evidence exists for a link between several other implicated kinases and COVID-19. MAPK11 and MAPK14 both belong to the p38 MAPK family which are involved in the cellular responses to extracellular stimuli including proinflammatory cytokines such as IL-6^40^. Previously, it has been shown that one of the proteins expressed by SARS-CoV virus upregulates p38 MAPK^41^. A global phosphorylation analysis of SARS-CoV-2 infected epithelial cells also identified targets of MAPK14 and MAPK11 as kinases upregulated during infection that make important contributions to host responses^35^. Accordingly, inhibition of MAPK14 and MAPK11 family has been proposed as a potential therapeutic approach in COVID-19^40^. In addition to being a downstream target of TLR-signaling pathways, including TLR3 in airway epithelial cells^42^, MAPK14 regulates IL-6 expression and mRNA stability^43^. IL-6 circulating levels are elevated in severe COVID-19^44^. Moreover, MAPK14 is an important signal transducer of IL-17 in endothelial cells^45^, involved in neutrophilic inflammation, that may be important mediators of thrombosis in COVID-19 via the release of Neutrophil-Extracellular-Traps^33,46^. MAPK14 is an important target of the potent corticosteroid dexamethasone^47^, that was shown to decrease mortality in severe COVID-19^48^. BMX and BTK are non-receptor tyrosine kinases that belong to the Tec kinase family. The Tec family has been shown to be involved in the intracellular signaling mechanisms of cytokine receptors and antigen receptor signaling in lymphocytes^49^. BMX has been shown to link both MYD88, another TLR-signaling adapter, and Focal Adhesion Kinases, a kinase associated with integrin activation, to the synthesis of IL-6^50^. This published evidence makes BMX and BTK candidate contributors to the cytokine release storm. Accordingly, the inhibition of genes in this family, and particularly BTK^43^, has been proposed as a therapeutic approach to protect COVID-19 patients against pulmonary injury^44^ and to block thrombo-inflammation^45^. Two potential downstream targets of BTK according to our regulatory networks (Fig. 3), Tumor necrosis factor ligand superfamily member 14 (TNFSF14) and XIAP-associated factor 1 (XAF1), have been identified in a single-cell transcriptomic study comparing IAV and SARS-CoV-2 responses^51^. TNFSF14 is a ligand of the lymphotoxin beta receptor that amplifies NFκB signaling in T lymphocytes to increase IFN-gamma production^52^. XAF1, as its name implies, binds XIAP (BIRC4), an important regulator of inflammatory signaling and apoptosis, that increases TRAIL-mediated apoptosis in response to IFNβ^53^. While these responses are likely desirable during early phases of the infection, whether they can also contribute to immunopathology in the second sustained phase of the disease warrants further investigation. While published data supports the current pipeline, it is expected that highly studied gene families such as JAK-STAT and p38 MAPK would feature more prominently, based on the greater availability of information on these kinases. One of the benefits of the current approach is to highlight the potential role of much less studied kinases that may nevertheless be important contributors following experimental validation. In this regard, the pseudokinase VRK3, which has not been studied in the context of human viral infections, can negatively regulate the MAPK ERK, known for its role in regulating inflammation downstream of the protein kinase Tumor Promoting Locus-2^54–56^. Another example is the top hit identified, MYO3A, a motor and kinase activity containing protein that can be found in actin bundle-based structures of cells^57^, for which no information is available in the context of infection or inflammation.

In this study, we focused on data corresponding to cell lines as opposed to patient data. The reason for this choice was the availability of data on several epithelial cell lines infected by different respiratory viruses generated by the same lab using an identical experimental procedure. Repeating the same analysis based on patient data requires a similarly consistent data acquisition setup corresponding to patients infected by different viruses; otherwise, the differences between experimental protocols and batch effects may introduce significant confounders in the analysis.

One of the limitations of this study was using only transcriptomic profiles of the samples in reconstructing the TRN. While we used ChIP-seq data for validation of our findings, the ChIP-seq data did not correspond to cells infected by SARS-CoV-2. A multi-omics approach that incorporates matched molecular profiles of the same set of samples can improve the analysis and identify other regulatory mechanisms involved in the response of host to SARS-CoV-2. As new datasets become available in this domain, we will apply a multi-omics variation of the proposed pipeline to achieve this goal. Another limitation of this study was the difficulty in validating the relationship between the implicated kinases and their target genes and TFs. While the LINCS dataset provides an extremely useful resource, the fact that the mRNA expressions of only 978 genes are measured as a result of kinase knockdowns, limited our ability to validate all our identified relationships. In spite of these limitations, we believe that building TRNs associated with the response of host to SARS-CoV-2, when compared against mechanisms common among other respiratory viruses, is essential in better understanding the biological processes involved in this infection. This study serves as the first step in achieving this goal.

In conclusion, the results obtained in this manuscript are the product of a novel data analysis pipeline that strings together three powerful computational tools (InPheRNo, KnowEnG GSC, and foRWaRD), which identified regulatory networks, pathways, and kinases, many of which have already been associated with COVID-19 in previous studies. These results also provided further information on putative regulatory mechanisms underpinning the infection of epithelial cells by SARS-CoV-2 and identified novel potential therapeutic targets that can serve as the basis for future identification and development of drugs that mitigate the impact of COVID-19 in individuals at risk of severe complications.

## Methods

### Data collection

We downloaded RNA-seq gene expression profiles of human lung epithelial cells infected by respiratory viruses from the Gene Expression Omnibus (GEO) database (accession number: GSE147507). More specifically, we used 33 samples corresponding to independent biological replicates of A549 cells infected with SARS-CoV-2, RSV, IAV, and HPIV3, NHBE cells infected with SARS-CoV-2, IAV, and IAVdNS, A549-ACE2 cells infected with SARS-CoV-2, and Calu3 cells infected with SARS-CoV-2.

We downloaded the list of human TFs from AnimalTFDB^58^. Experimentally verified protein–protein interaction network from the STRING database^27^ and HumanNet integrated network^34^ were downloaded from KnowEnG’s knowledge network (version 17.06) available at the address https://github.com/KnowEnG/KN_Fetcher/blob/master/Contents.md. The list of target genes for the top TFs (identified using ChIP-seq) was downloaded from the GTRD database (http://gtrd.biouml.org/downloads/20.06/intervals/target_genes/Homo%20sapiens/genes%20promoter%5b-1000,+100%5d/). In this dataset, a gene is considered to be a target of a TF if its promoter region (defined as the interval [− 1000, + 100] bp relative to gene’s transcriptional start site) contains at least one GTRD meta-cluster for the TF. The meta-clusters reflect ChIP peaks for the same TF-gene but integrated from different experimental conditions and peak-calling methods^25^. We used this dataset since it uses a consistent and principled computational pipeline to integrate ChIP-seq data from many different studies and, to the best of our knowledge, is the most comprehensive database of such regulatory evidence.

To form the aggregated KSI network, we obtained kinase-substrate relationships from three previous studies. The interactions corresponding to homo sapiens were downloaded from PhosphoSitePlus database (http://www.phosphosite.org)^30^, PhosphoNetworks (http://www.phosphonetworks.org)^31^, and the supplementary material of an independent study^32^. After removing duplicate edges, we formed a KSI involving 29,594 kinase-substrate relationships corresponding to 406 unique kinases and 3942 unique substrates. LINCS Level 5 consensus signatures (‘trt_sh.cgs’) corresponding to shRNA knockdowns in A549 cell line were obtained from GEO with the accession number (GSE92742). We used the Consensus Gene Signatures (CGS) data since they correspond to gene expression changes that are common among multiple shRNAs that target the same gene, mitigating off-target effects^59^.

### Reconstruction of SvOV TRN using InPheRNo

InPheRNo^18^ is a computational method that utilizes a probabilistic graphical model to combine information on the significance (pseudo p-value) of gene-TF associations (from their expression profiles) with information on the significance (p-value) of gene-phenotype associations to construct phenotype-relevant TRNs. As input, it accepts a list of TFs, the expression profiles of genes and TFs (in multiple samples), and the p-value of association between genes’ expression and a phenotype.

To construct the SvOV TRN using InPheRNo, we first performed differential expression analysis using EdgeR^20^ with the cell type and the time of measurement post-infection as confounding factors. Next, we quantile normalized the gene expression profiles using voom-limma^60^ and then z-score normalized the results. We ran InPheRNo (downloaded from https://github.com/KnowEnG/InPheRNo) with 1000 iterations, 500 repeats and default values for other parameters.

### Pathway enrichment analysis using KnowEnG’s gene set characterization pipeline

We used KnowEnG’s gene set characterization (GSC) pipeline^26^ (http://www.knoweng.org/analyze) to perform pathway enrichment analysis. For the standard mode of GSC pipeline (without the use of any gene interaction network), we chose Reactome pathways^28^ as the option for target pathway collection and the rest of parameters were left as default. We excluded pathways with smaller than 10 genes and adjusted the enrichment p-values (Fisher’s exact test) for multiple tests using Benjamini–Hochberg false discovery rate (FDR). Pathways with FDR < 0.05 were considered statistically significant.

The network-guided mode of this pipeline is an implementation of an algorithm called DRaWR^61^, which utilizes random walk with restarts (RWR) algorithm on a user-selected gene interaction network to rank pathways based on their relevance to a query gene-set. Including a gene interaction network (e.g., a protein–protein interaction (PPI) network) enriches the analysis and enables identification of important pathways that may not be detectable using simple overlap-based Fisher’s exact test. For this mode of the GSC pipeline, we used ‘STRING Experimental PPI’ option for the knowledge network (which corresponds to experimentally verified protein–protein interaction edges from the STRING database^27^) and the amount of network smoothing was set to the default 50%. Reactome was used as the target pathway collection. The network-guided mode of this pipeline does not provide a p-value for the pathways, since the task of identification of pathways is done using an algorithm called DRaWR^61^, which is based on random walk with restarts (RWR) on graphs (instead of a statistical test). Instead, it provides a score for each pathway called the ‘difference score’. Difference score values larger than 0.5 correspond to pathways that have a higher steady-state RWR probability when the input query gene set is used as the restart set, compared to their steady-state probability when the query gene set is not used as the restart set in the random walk. The difference scores ensure that a pathway is not simply scored high due to the network bias and having many edges in the network.

For pathway enrichment analysis of the set of top TFs, the universe (i.e., population) was considered to be the set of all TFs present in our study. For pathway enrichment analysis of a TF and its target genes, the universe was considered to be the set of all genes and TFs present in our study.

### Ranking kinases using foRWaRD

To use foRWaRD to rank kinases associated with the SvOV network, we provided it with the aggregated KSI, the HumanNet integrated network^34^, and a query set containing the top TFs obtained from the SvOV TRN (21 TFs). foRWaRD first forms a heterogenous network by superimposing the substrates on their corresponding gene nodes in the gene–gene interaction network. Then, it performs two runs of the RWR algorithm on this heterogenous network: one run with the query set (set of TFs) as the restart set of the RWR and another run with all nodes as the restart set (to be used as control). Each run of the RWR provides a probability score for each node (including those corresponding to kinases), representing the relevance of the node to the restart set. Finally, a normalized score for each kinase is obtained by comparing the scores of the two runs of the RWR, and kinases are ranked based on how much their query set score is higher than their background (i.e., control) score.

## Supplementary Information

Supplementary Figure S1.

Supplementary Table S1.

Supplementary Table S2.

Supplementary Table S3.

Supplementary Table S4.

Supplementary Table S5.

Supplementary Table S6.

Supplementary Table S7.

Supplementary Table S8.

## Data Availability

All the datasets used in this study are publicly available as detailed in “Data collection” section of “Methods”. The data generated in this study are included in this published article [and its supplementary information files].
