# Supplementary material for: Identification of transcriptional regulatory network associated with response of host epithelial cells to SARS-CoV-2
Source: Sci Rep. 2021 Dec 14;11:23928. doi: 10.1038/s41598-021-03309-5 (PMC8671548; doi:10.1038/s41598-021-03309-5)
Supplement: Supplementary file 1 — Supplementary Figure S1. [file 41598_2021_3309_MOESM1_ESM.pdf]

Supplementary Figure

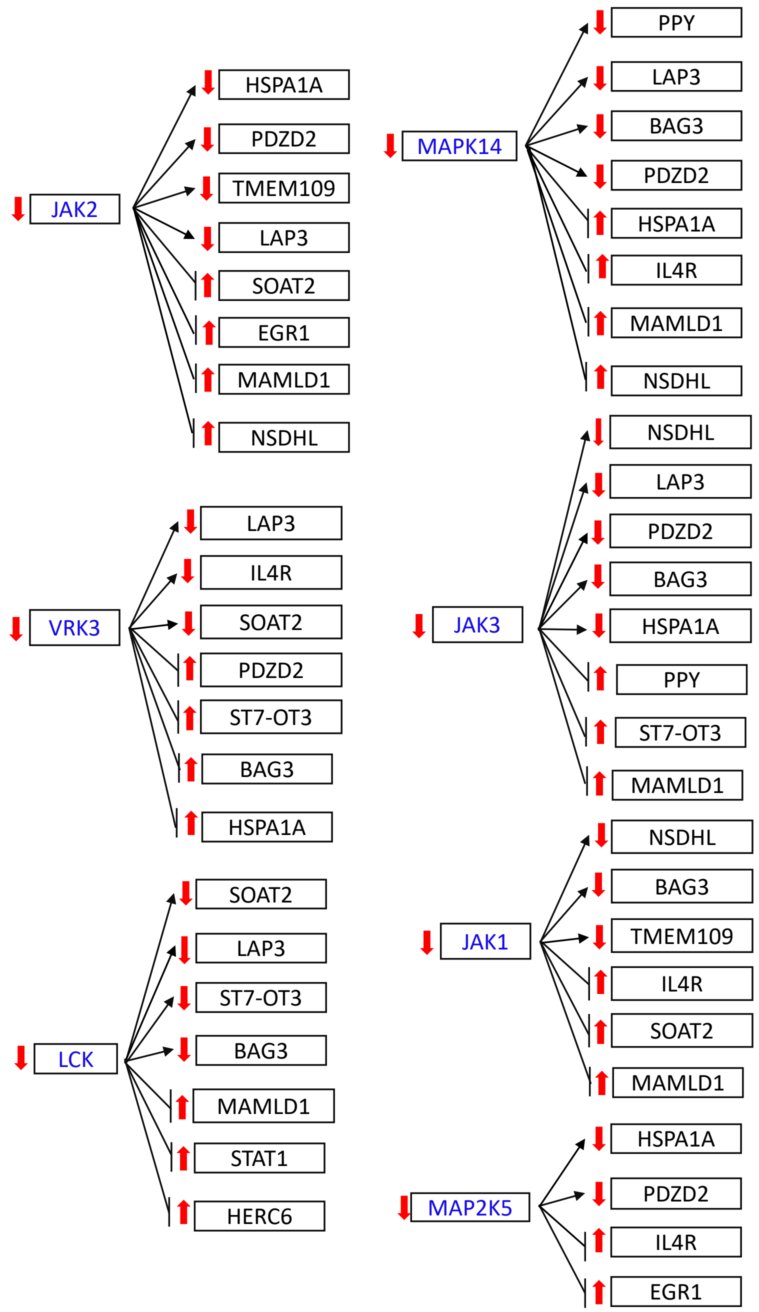

Figure S1: The effect of kinase knockdowns on genes and TFs shared between the SvOV TRN and L1000 landmark genes. An upward arrow shows that the expression of the gene in A549 cells increased and the change in the expression was among the top 15% of all landmark genes. A downward arrow shows that the expression of the gene in A549 cells decreased and the change in the expression was among the bottom 15% of all landmark genes.
